# Identification and validation of the molecular subtype and prognostic signature for clear cell renal cell carcinoma based on neutrophil extracellular traps

**DOI:** 10.3389/fcell.2022.1021690

**Published:** 2022-11-29

**Authors:** Jing Quan, Banggao Huang

**Affiliations:** Urology & Nephrology Center, Department of Urology, Zhejiang Provincial People’s Hospital, Affiliated People’s Hospital, Hangzhou Medical College, Hangzhou, China

**Keywords:** clear cell renal cell carcinoma, neutrophil extracellular traps, prognostic signature, molecular subtype, ceRNA

## Abstract

**Background:** Renal cell carcinoma (RCC) is one of the most common cancers, with an annual incidence of nearly 400,000 cases worldwide. Increasing evidence has also demonstrated the vital role of neutrophil extracellular traps (NETs) in cancer progression and metastatic dissemination.

**Methods:** Consensus cluster analysis was performed to determine the number of ccRCC subtypes. The Kruskal–Wallis test or Student t-test was performed to evaluate the difference of infiltrating immune cell and gene expression in different groups. The Kaplan–Meier (KM) method was used to draw the survival curve. LASSO cox regression analysis was conducted to construct a NET-related prognostic signature. We also constructed a lncRNA–miRNA–mRNA regulatory axis by several miRNA and lncRNA target databases.

**Results:** A total of 23 differentially expressed NET-related genes were obtained in ccRCC. Three clusters of ccRCC cases with significant difference in prognosis, immune infiltration, and chemotherapy and targeted therapy were identified. LASSO Cox regression analysis identified a NET-related prognostic signature including six genes (*G0S2*, *DYSF*, *MMP9*, *SLC22A4*, *SELP*, and *KCNJ15*), and this signature had a good performance in predicting the overall survival of ccRCC patients. The expression of prognostic signature genes was significantly correlated with the pTMN stage, immune infiltration, tumor mutational burdens, microsatellite instability, and drug sensitivity of ccRCC patients. *MMP9* was identified as the hub gene. We also identified the lncRNA UBA6-AS1/miR-149-5p/MMP9 regulatory axis for the progression of ccRCC.

**Conclusion:** Collectively, the current study identified three molecular clusters and a prognostic signature for ccRCC based on neutrophil extracellular traps. Integrative transcriptome analyses plus clinical sample validation may facilitate the biomarker discovery and clinical transformation.

## 1 Introduction

Renal cell carcinoma (RCC) is one of the most common cancers, with an annual incidence of nearly 400,000 cases worldwide (Gray and Harris, 2019; Chowdhury et al., 2020). In the past several years, there was a notable increase in the incidence of RCC (Chowdhury et al., 2020). Despite several risk factors being identified for the development of RCC, including the alteration of *VHL* and *HIF* genes, the specific mechanisms have not been fully elucidated (Deleuze et al., 2020). Significant advances had been made in the therapy and management of RCC over the past two decades, which largely improved the prognosis (Motzer et al., 2007). Recent studies have also suggested that molecular subtype analysis and discrimination of different characteristics of RCC patients could achieve precise treatment (Su et al., 2015; Riazalhosseini and Lathrop, 2016; Ricketts et al., 2018). However, the 5-year survival for patients with advanced ccRCC remained very poor (Zeng et al., 2019). No ideal prognostic biomarker or signature has been identified for the prognosis of RCC clinically.

Neutrophil extracellular traps (NETs) were composed of depolymerized chromatin and intracellular granule proteins released by activated neutrophils (Rada, 2019). The formation of NETs was accompanied by the death of neutrophils, called NETosis, which was distinct from apoptosis and necrosis (Yipp and Kubes, 2013; Vorobjeva and Chernyak, 2020). NETs were also involved in many diseases, including rheumatoid arthritis, thrombosis, cardiovascular diseases, and cancer (Bonaventura et al., 2020; Masucci et al., 2020). Increasing evidence has also demonstrated the vital role of NETs in cancer progression and metastatic dissemination (Huang et al., 2020; Masucci et al., 2020). Moreover, NETosis and systemic lymphocyte perturbations played a vital role in the tumor progression of localized RCC with tumor thrombus (Shang et al., 2021). However, the specific role of NETs in the development and prognosis of RCC has not been fully clarified. In our study, bioinformatics analysis was performed to explore the expression patterns, prognostic values, and potential regulatory axes of NET-related genes in RCC.

## 2 Materials and methods

### 2.1 Data source and preprocessing

Based on previous studies, a total of 69 NET-related genes (Supplementary Table S1) were obtained (Şenbabaoğlu et al., 2016; Papayannopoulos, 2018; Zhang et al., 2022). The gene expression profile of ccRCC (*n* = 524) was downloaded from TCGA (https://portal.gdc.cancer.gov/) database on 18 March 2022. The mRNA expression data were then normalized into the transcripts per million (TPM) value before further analysis. The differentially expressed NET-related genes were screened using the “limma” package with “*p* < 0.01 and Log2 |(Fold Change)| >2” as the threshold. The genetic mutation data on ccRCC including single-nucleotide variants (SNVs) and copy number variation (CNV) were isolated from TCGA *via* the UCSC Xena server (https://xena.ucsc.edu/). The ccRCC dataset from the International Cancer Genome Consortium (ICGC) was used as the validation set.

### 2.2 Genetic mutation and functional enrichment analysis

The SNV landscape was drawn using the “maftools” package. The location of CNV alteration on chromosomes was identified using the “RCircos” package. Using the “ clusterProfiler ” package, we then performed Gene Ontology (GO) and Kyoto Encyclopedia of Genes and Genomes (KEGG) pathway analyses with “*p* < 0.05” as the threshold.

### 2.3 Consensus cluster analysis

The consensus clustering analysis could provide quantitative and visual stability evidence for estimating the number of cancer subtypes (Wilkerson and Hayes, 2010; Li et al., 2020). The optimum subtype of TCGA ccRCC was determined with the “ConsensusClusterPlus” package (Wilkerson and Hayes, 2010). The Kaplan–Meier (KM) method was used to draw the survival curve of ccRCC in each cluster. “CIBERSORT” algorithms were used to calculate the level of infiltrating immune cells of the ccRCC case (Chen et al., 2018). Using the “ggplot 2” package, we performed the Kruskal–Wallis test to evaluate the difference of infiltrating immune cells between each cluster. The chemotherapeutic response for each cluster was evaluated using the “pRRophetic” package.

### 2.4 Identification of prognostic biomarkers and prognostic signature construction

The KM method was used to draw the overall survival (OS) curve, progression free survival (PFS) curve, and disease-specific survival (DSS) curve. Based on the results of OS, PFS, and DSS, we used the “glmnet” package to perform the least absolute shrinkage and selection operator (LASSO) regression analysis with 10-fold cross-validation, which could identify candidate genes and construct a prognostic signature. The risk score of cRCC cases = Σi(Coefi·Expi) (*Coef* is the coefficient, and *Exp* is the gene expression). The KM method was also used to draw the 3-year/5-year OS curve of ccRCC in the high-/low-risk group. We also drew the ROC curve with the “survivalROC” R package for analyzing the performance of this prognostic signature in ccRCC.

### 2.5 Prognostic signature gene analysis

The ESTIMATE algorithm was used to evaluate the tumor mutational burden (TMB) and Microsatellite Instability (MSI) scores of ccRCC (Liu et al., 2020). Spearman’s correlation analysis was conducted to calculate the correlation coefficient between prognostic signature genes and the abundance of immune cells from TIMER (https://cistrome.shinyapps.io/timer/) (Li et al., 2017). The IC_50_ values of 481 small molecules in 1,001 cell lines and there corresponding gene expression were isolated from the Genomics of Therapeutics Response Portal (CTRP). Pearson correlation analysis was conducted to calculate the correlation coefficient between gene expression and drug IC_50_ concentration. The Kruskal–Wallis test or Student t-test was performed to analyze the difference of gene expression in different groups of ccRCC. We then identified the hub gene using STRING (https://string-db.org/). TTo explore the miRNA targets of the hub gene, three miRNA target prediction databases, miRDB (http://mirdb.org/), TargetScan (https://www.targetscan.org/) and miRWalk (http://mirwalk.umm.uni-heidelberg.de/)] were used. We also detected lncRNA targets interacting with miRNA using LncBase (http://carolina.imis.athena-innovation.gr/) and StarBase (http://starbase.sysu.edu.cn/).

## 3 Results

### 3.1 Expression and mutation landscape of neutrophil extracellular trap-related genes in ccRCC

**FIGURE 1:**
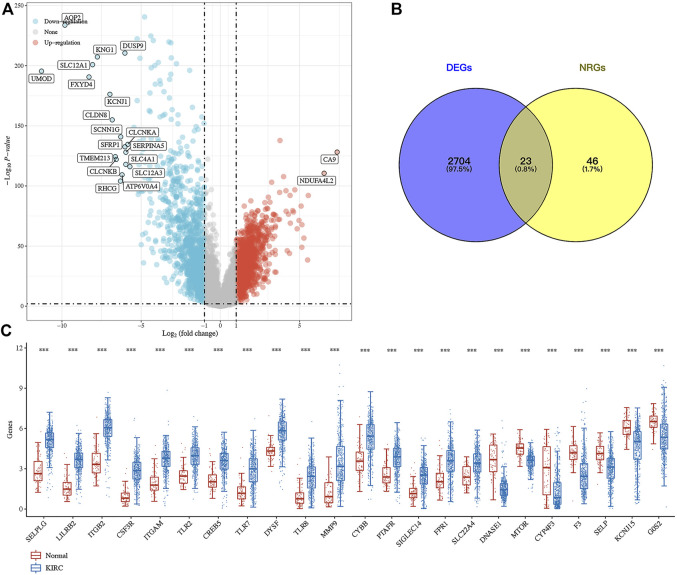
Expression landscape of neutrophil extracellular trap-related genes in ccRCC. **(A)** Volcano plot about the differentially expressed genes in ccRCC. **(B–C)** Venn diagram about the number of differentially expressed neutrophil extracellular trap-related genes in ccRCC. ***p < 0.001; ccRCC, clear cell renal cell carcinoma.

**FIGURE 2:**
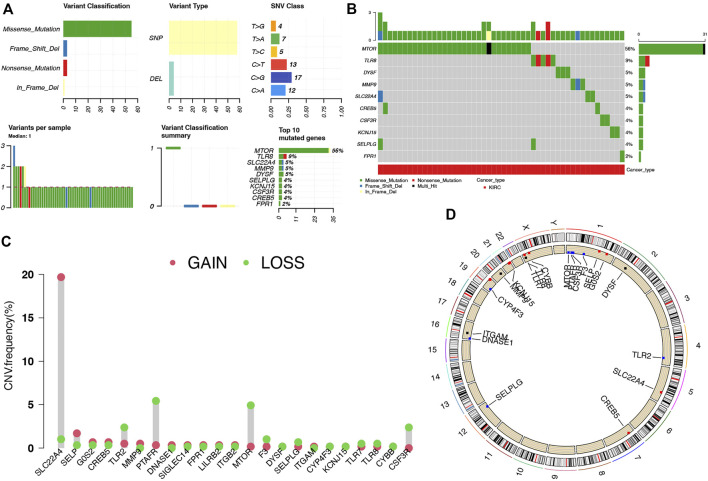
Landscape of genetic mutation of NET-related genes in ccRCC. **(A–B)** SNV frequency and classification of NET-related genes in LUAD. **(C–D)** CNV alteration of NET-related genes in ccRCC and their location on chromosomes. NETs, neutrophil extracellular traps; ccRCC, clear cell renal cell carcinoma.

Compared with normal kidney tissues, a total of 1,427 genes were upregulated and 1,300 genes were downregulated in ccRCC (Figure 1A, *p* < 0.01). Among these genes, 23 were differentially expressed NET-related genes (Figure 1B). To be more specific, the expression of NET-related genes *SELPLG*, *LILRB2*, *ITGB2*, *CSF3R*, *ITGAM*, *TLR2*, *CREB5*, *TLR7*, *DYSF*, *TLR8*, *MMP9*, *CYBB*, *PTAFR*, *SIGLEC14*, *FPR1*, and *SLC22A4* was upregulated, while the expression of *DNASE1*, *MTOR*, *CYP4F3*, *F3*, *SELP*, *KCNJ15*, and *G0S2* was downregulated in ccRCC (Figure 1C, *p* < 0.001). The SNV landscape of 23 differentially expressed NET-related genes in ccRCC is shown in Figures 2A,B, which reveals that the most frequently mutated gene was mTOR, followed by TLR8. Missense mutation and C>G ranked the main variant classification and SNV class, respectively (Figure 2A). Among the 23 differentially expressed NET-related genes, most of them had copy number deletion, and SELPLG and SELP had copy number amplification (Figure 2C). The location of these NET-related genes on chromosomes is shown in Figure 2D.

### 3.2 Functional enrichment analysis

GO and KEGG pathway analyses were performed using 23 differentially expressed NET-related genes. As a result, these genes were mainly involved in myeloid leukocyte activation, immune response, positive regulation of interleukin-6 production, neutrophil-mediated immunity, pattern recognition receptor activity, and immune receptor activity in GO analysis (Supplementary Figure S1A). Moreover, KEGG pathway analysis revealed that these genes were mainly associated with the neutrophil extracellular trap formation, leukocyte transendothelial migration, PI3K-Akt signaling pathway, and Toll-like receptor signaling pathway (Supplementary Figure S1B).

### 3.3 Consensus clustering identified three clusters of ccRCC

**FIGURE 3:**
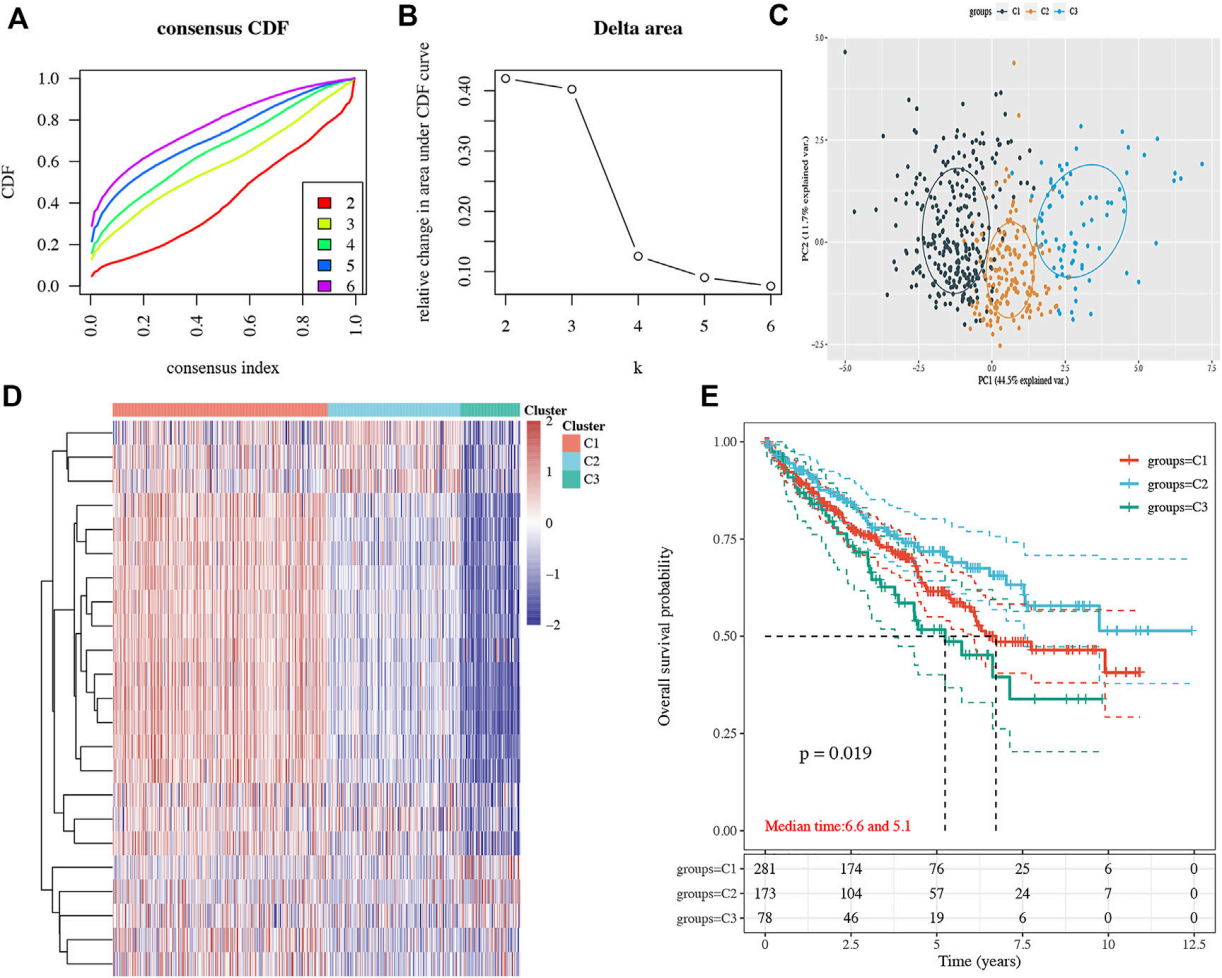
Subtype analysis in ccRCC based on NET-related genes. **(A–B)** CDF and delta area under the CDF curve in consensus clustering analysis. **(C–D)** Three subtypes of TCGA ccRCC cases were identified according to the consensus clustering matrix. **(E)** Overall survival curve of each cluster of ccRCC patients. ccRCC, clear cell renal cell carcinoma. CDF, cumulative distribution function.

**FIGURE 4:**
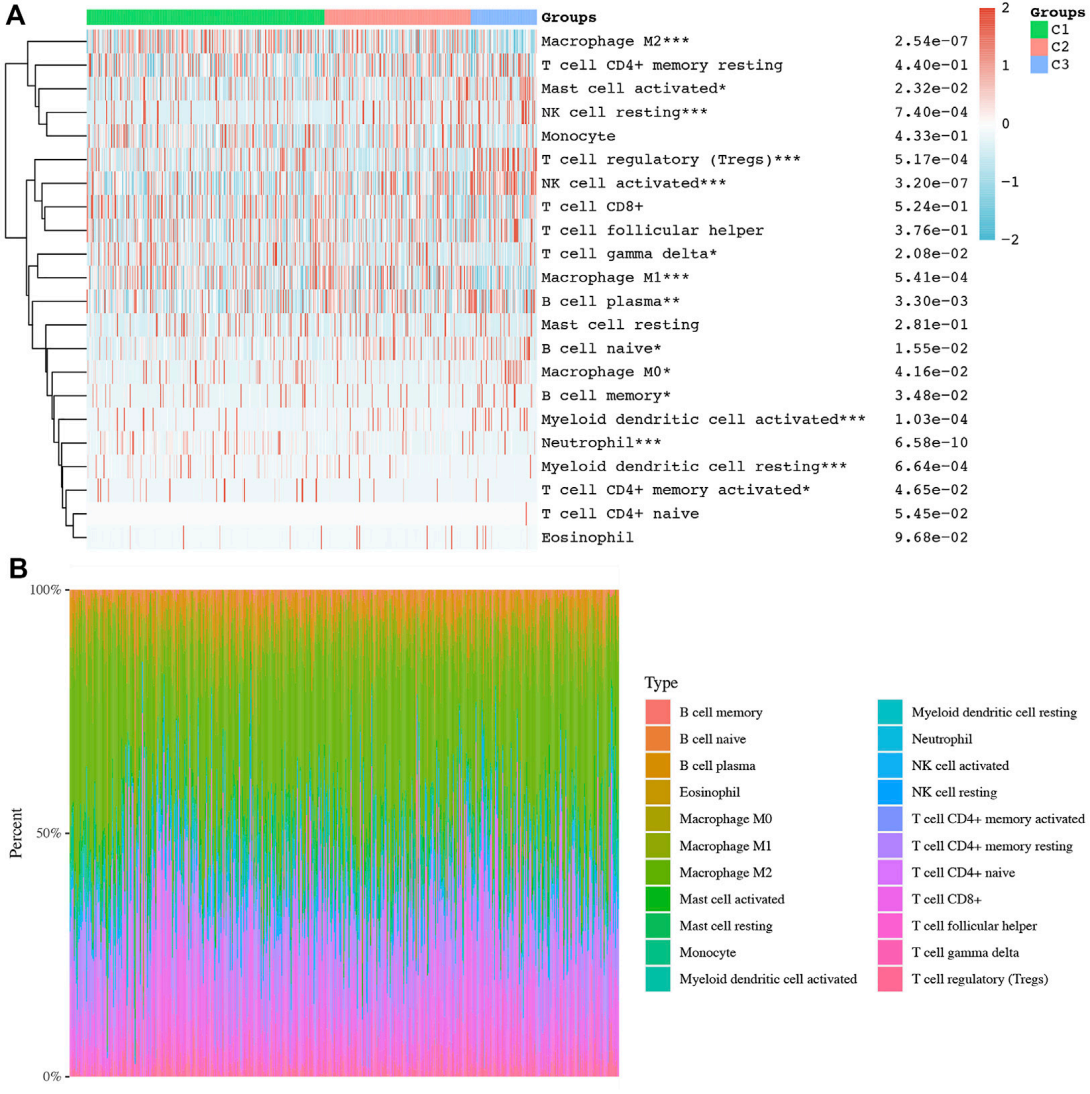
Difference of immune cell infiltration in three subtypes of ccRCC. **(A)** Abundance of different immune cells in three clusters of ccRCC. **(B)** Distribution of different immune cells in each ccRCC case. *p < 0.05; **p < 0.01; ***p < 0.001; ccRCC, clear cell renal cell carcinoma.

**FIGURE 5:**
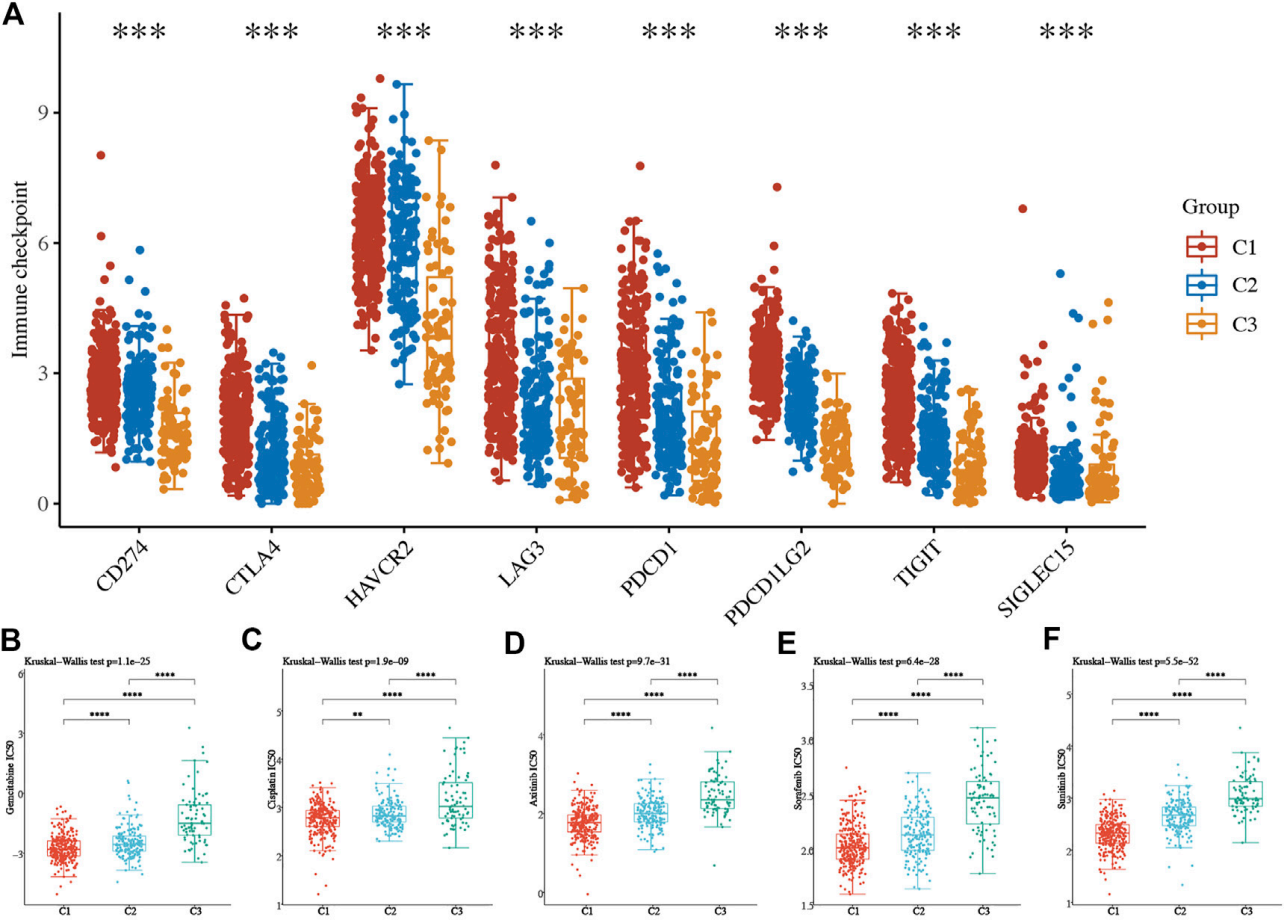
Difference of immune checkpoints and the IC_50_ score in three subtypes of ccRCC. **(A)** Expression of immune checkpoints in three subtypes of ccRCC. **(B–F)** The IC_50_ score of common drugs of each cluster of ccRCC patients. *p < 0.05; **p < 0.01; ***p < 0.001; ccRCC, clear cell renal cell carcinoma.

Consensus clustering analysis was conducted to cluster ccRCC patients based on 23 differentially expressed NET-related genes. According to CDF values and the delta area, we determined three clusters of TCGA-ccRCC patients (Figures 3A–D). Among these three clusters of ccRCC patients, cluster 2 had the best OS rate, while cluster 3 had the worst OS rate (Figure 3E, *p* = 0.019). As immunotherapy was considered as one of the most promising therapeutic strategies for ccRCC patients in the advanced stage (Zhou et al., 2020; Bi et al., 2021), we then evaluated the difference of three clusters in immune infiltration. Interestingly, significant difference was obtained in the level of naïve B cells, memory B cells, plasma B cells, CD4^+^ memory-activated T cells, regulatory T cells(Tregs), gamma delta T cells, resting NK cells, activated NK cells, M0 macrophages , M1 macrophages, M2 macrophages , resting myeloid dendritic cells, activated myeloid dendritic cells, activated mast cells, and neutrophils among these three clusters (Figure 4A). The percentage of each immune cell in ccRCC is shown in Figure 4B. Moreover, the data also suggested a higher expression of immune checkpoints in cluster 1 than that in cluster 2/3 (Figure 5A, all *p* < 0.001). We also analyzed the IC_50_ value of common chemotherapeutic drugs and targeted therapeutics in three clusters. As expected, cluster 3 had a higher IC_50_ value of gemcitabine (Figure 5B, *p* = 1.1e-25), cisplatin (Figure 5C, *p* = 1.95–9), axitinib (Figure 5D, *p* = 9.7e-31), sorafenib (Figure 5E, *p* = 6.4e-28), and sunitinib (Figure 5F, *p* = 5.5e-52) than cluster 1/2.

### 3.4 The prognostic significance of neutrophil extracellular trap-related genes in ccRCC

**FIGURE 6:**
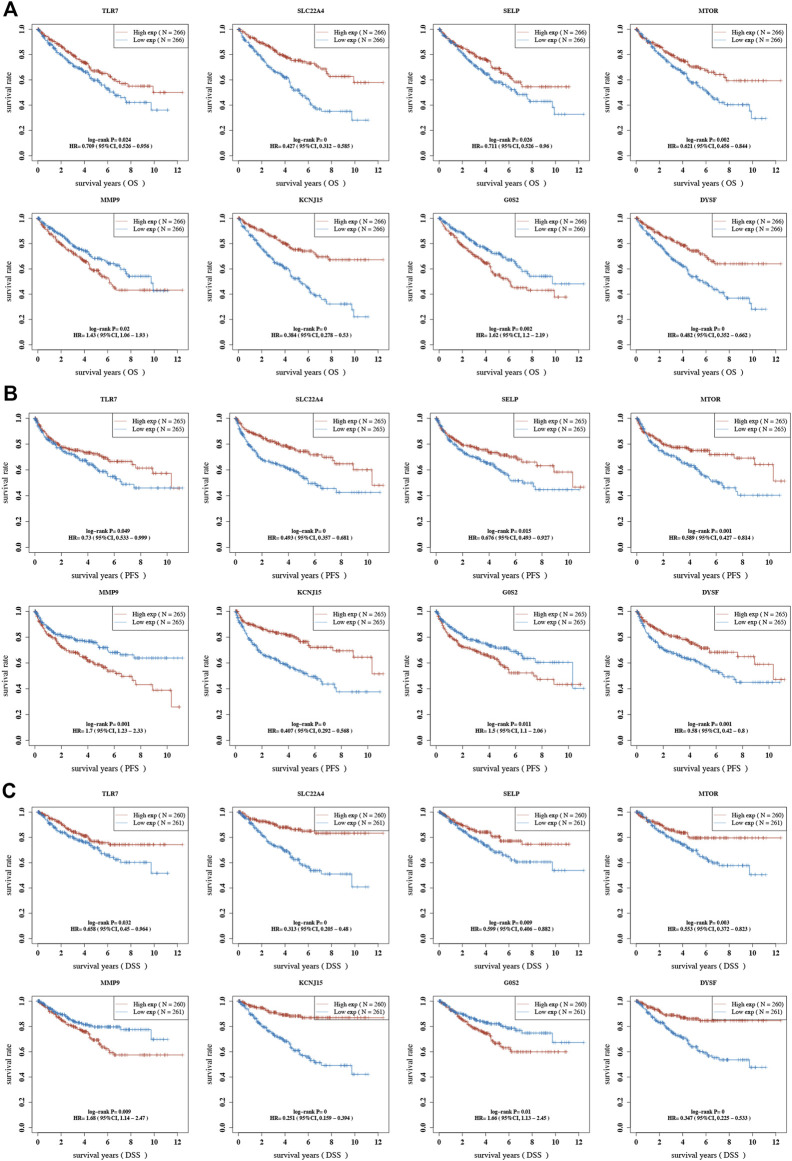
Prognostic value of NET-related genes in ccRCC. The Kaplan–Meier curve revealed the result of overall survival **(A)**, progression-free survival **(B)**, and disease-specific survival **(C)** of NET-related genes in ccRCC. NETs, neutrophil extracellular traps; ccRCC, clear cell renal cell carcinoma.

The result of OS analysis revealed that ccRCC patients with high levels of *G0S2* and *MMP9* and low levels of *CREB5*, *SELP*, *TLR7*, *CYBB*, *TLR8*, *MTOR*, *DYSF*, *SLC22A4*, and *KCNJ15* had a poor OS rate (Figure 6A and Table 1). Moreover, high levels of *MMP9*, *G0S2*, and *F3* and low levels of *TLR7*, *SELP*, *MTOR*, *DYSF*, *SLC22A4*, and *KCNJ15* were associated with a poor PFS rate in ccRCC (Figure 6B and Table 2). As for DSS analysis, the result suggested a poor clinical outcome in ccRCC patients with high levels of *MMP9*, *G0S2*, *F3* and low levels of *TLR7*, *TLR8*, *CREB5*, *CYBB*, *SELP*, *MTOR*, *DYSF*, *SLC22A4*, and *KCNJ15* (Figure 6C and Table 3). Due to the significant role of *TLR7*, *DYSF*, *MMP9*, *SLC22A4*, *MTOR*, *SELP*, *KCNJ15*, and *G0S2* in OS, PFS, and DSS analyses (Figures 6A–C), we suggested *TLR7*, *DYSF*, *MMP9*, *SLC22A4*, *MTOR*, *SELP*, *KCNJ15*, and *G0S2* as potential prognostic biomarkers for ccRCC**.**

**TABLE 1:** NET-related genes with significant prognosis in overall survival analysis.

Gene	*p*-value	HR	Low 95% CI	High 95% CI
*CREB5*	0.044	0.735	0.545	0.991
*TLR7*	0.024	0.709	0.526	0.956
*DYSF*	<0.001	0.482	0.352	0.662
*TLR8*	0.013	0.682	0.505	0.921
*MMP9*	0.020	1.430	1.059	1.932
*CYBB*	0.016	0.690	0.511	0.932
*SLC22A4*	<0.001	0.427	0.312	0.585
*MTOR*	0.002	0.621	0.456	0.844
*SELP*	0.026	0.711	0.526	0.960
*KCNJ15*	<0.001	0.384	0.278	0.530
*G0S2*	0.002	1.620	1.196	2.194

**TABLE 2:** NET-related genes with significant prognosis in progression-free survival analysis.

Gene	*p-*value	HR	Low 95% CI	High 95% CI
*TLR7*	0.049	0.730	0.533	0.999
*DYSF*	0.001	0.580	0.420	0.800
*MMP9*	0.001	1.695	1.232	2.332
*SLC22A4*	<0.001	0.493	0.357	0.681
*MTOR*	0.001	0.589	0.427	0.814
*F3*	0.018	1.461	1.066	2.001
*SELP*	0.015	0.676	0.493	0.927
*KCNJ15*	<0.001	0.407	0.292	0.568
*G0S2*	0.011	1.505	1.098	2.064

**TABLE 3:** NET-related genes with significant prognosis in disease-specific survival analysis.

Gene	*p*-value	HR	Low 95% CI	High 95% CI
*CREB5*	0.022	0.637	0.434	0.936
*TLR7*	0.032	0.658	0.450	0.964
*DYSF*	<0.001	0.347	0.225	0.533
*TLR8*	0.025	0.645	0.440	0.945
*MMP9*	0.009	1.677	1.138	2.472
*CYBB*	0.016	0.625	0.425	0.918
*SLC22A4*	<0.001	0.313	0.205	0.480
*MTOR*	0.003	0.553	0.372	0.823
*F3*	0.023	1.558	1.064	2.283
*SELP*	0.009	0.599	0.406	0.882
*KCNJ15*	<0.001	0.251	0.159	0.394
*G0S2*	0.010	1.665	1.132	2.450

### 3.5 Development of a prognostic signature for ccRCC based on neutrophil extracellular trap-related genes

**FIGURE 7:**
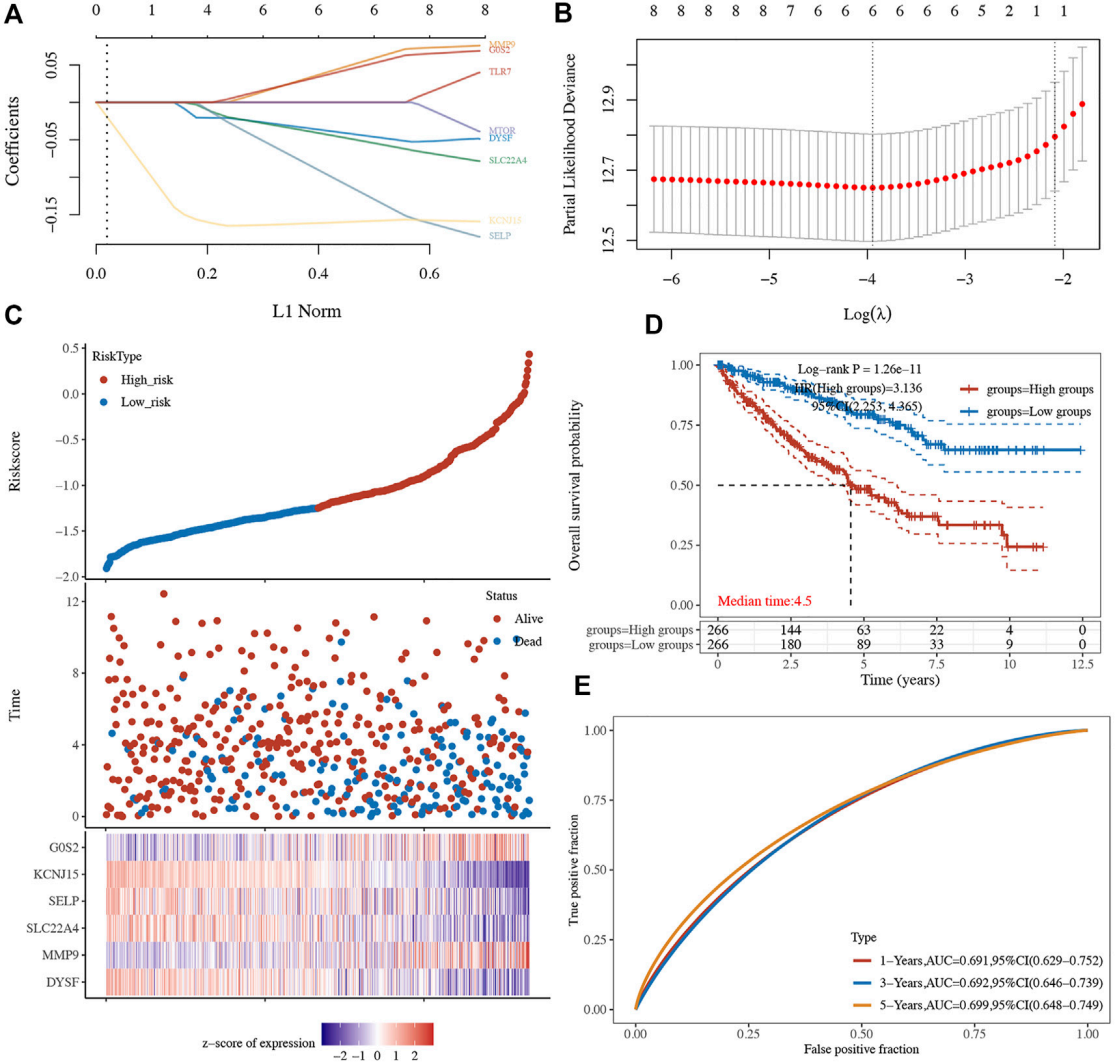
Prognostic signature in ccRCC based on NET-related genes in TCGA dataset. **(A–B)** The coefficient and partial likelihood deviance of the prognostic signature. **(C)** Riskscore distribution, patients’ survival status, and gene expression profile of the prognostic signature. **(D)** ccRCC patients in the high-risk group had a poor OS rate compared to that in the low-risk group. **(E)** AUCs of 1-year, 3-year, and 5-year ROC curves were 0.691, 0.692, and 0.699, respectively. NETs, neutrophil extracellular traps; ccRCC, clear cell renal cell carcinoma.

Based on the eight aforementioned potential prognostic biomarkers, we performed LASSO Cox regression analysis. As a result, six NET-related genes including *G0S2*, *DYSF*, *MMP9*, *SLC22A4*, *SELP*, and *KCNJ15* remained in this prognostic signature. The coefficient and partial likelihood deviance of prognostic signature is shown in Figures 7A,B. The riskscore of each ccRCC case was calculated using the following formula: Riskscore = (−0.0454)*DYSF + (0.0576)*MMP9 + (−0.0536)*SLC22A4 + (−0.1269)*SELP + (-0.1586)*KCNJ15 + (0.0513)*G0S2. Figure 7C showed the riskscore and survival status of RCC patients, and gene expression of the prognostic signature. Based on the riskscore, ccRCC cases were divided into two groups, and patients in the high-risk group had a poor OS rate compared with those in the low-risk group (Figure 7D, *p* = 1.26e-11). Moreover, the AUC of 1-year, 3-year, and 5-year ROC curves were 0.691, 0.692, and 0.699, respectively (Figure 7E), suggesting that this prognostic signature had a good performance in predicting the prognosis of ccRCC. The ICGC dataset was used as the validation set, and similar results were obtained (Supplementary Figures 2A–C). Further immune infiltration analysis demonstrated a significant negative correlation between the riskscore and the immune infiltration level of B cells, CD4^+^ T cells, CD8^+^ T cells, neutrophils, and macrophages (Supplementary Figures 3A–F).

### 3.6 Prognostic signature gene analysis

**FIGURE 8:**
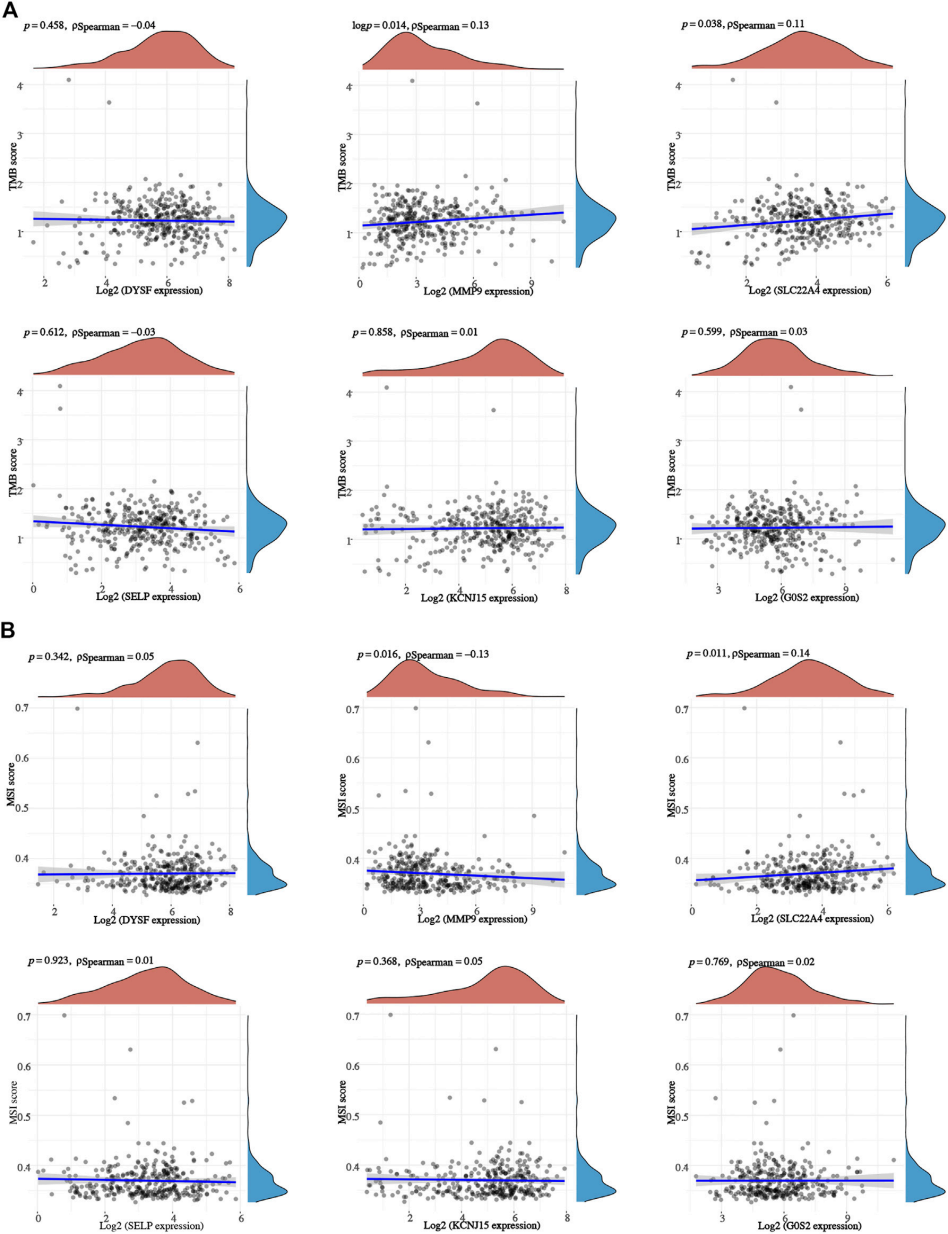
TMB/MSI analysis of prognostic signature genes in ccRCC. The correlation between prognostic signature gene expression and TMB **(A)** /MSI **(B)** score in ccRCC. ccRCC, clear cell renal cell carcinoma; TMB, tumor mutational burden; MSI, microsatellite instability (MSI).

**FIGURE 9:**
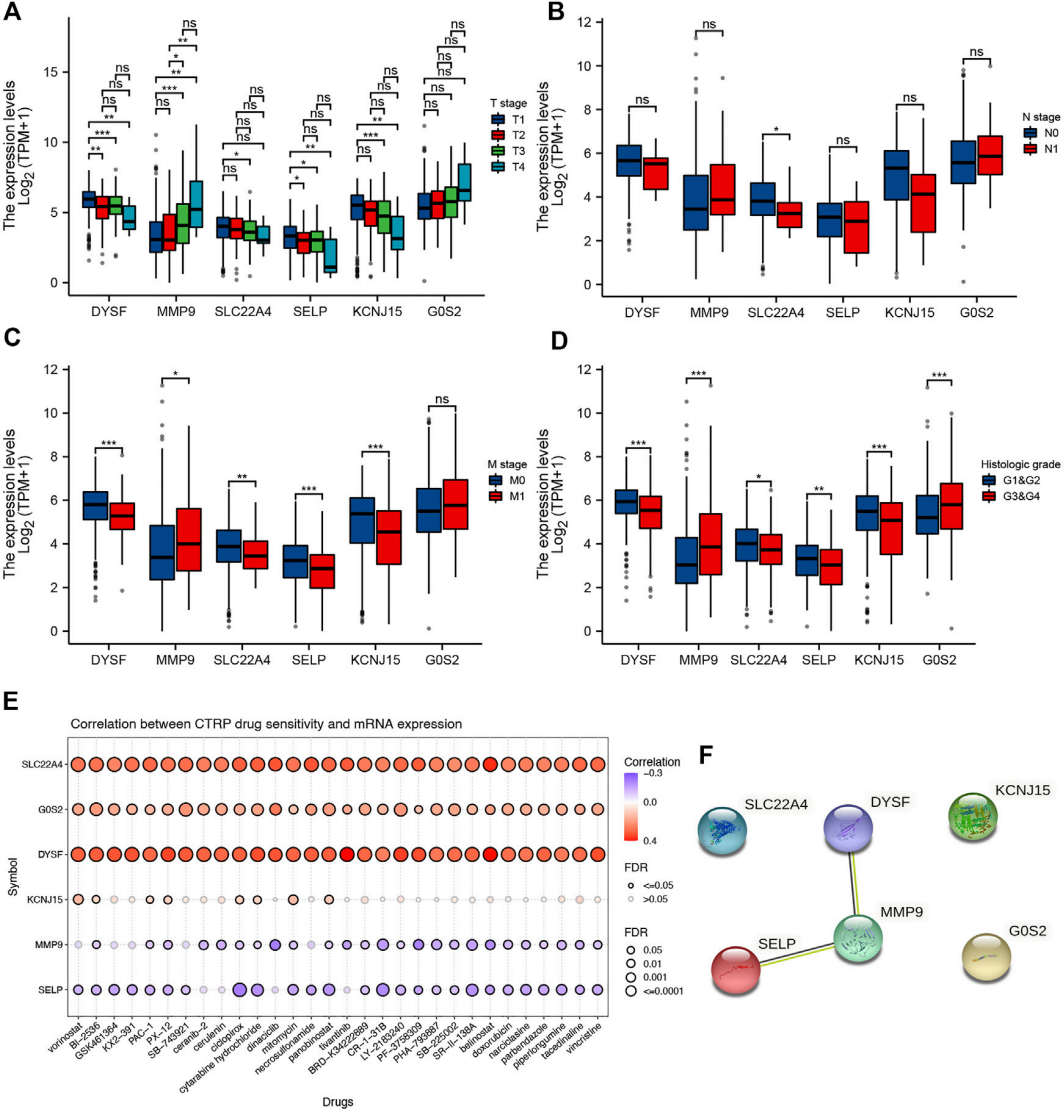
Prognostic signature gene analysis. Correlation between the pT stage **(A)**, pN stage **(B)**, pM stage **(C)**, tumor grade **(D)**, and the expression of prognostic signature genes in ccRCC. **(E)** Correlation between drug sensitivity and the expression of prognostic signature genes in ccRCC. **(F)** Protein–protein interaction network identified MMP9 as the hub gene among prognostic signature genes. *p < 0.05; **p < 0.01; ***p < 0.001; ccRCC, clear cell renal cell carcinoma.

We also analyzed the correlation between prognostic signature genes and immune cell infiltration. As a result, significant positive correlation was obtained between the expression of DYSF (Supplementary Figure 4A) and SELY (Supplementary Figure 4D) and the level of CD4^+^ T cells, CD8^+^ T cells, neutrophils, macrophages, and dendritic cells (all *p* < 0.05). As *MMP9* expression increased, the level of B cells, CD4^+^ T cells, neutrophils, macrophages, and dendritic cells increased (Supplementary Figure 4B). Moreover, the expression levels of *SLC22A4* (Supplementary Figure 4C), *KCNJ15* (Supplementary Figure 4E), and *G0S2* (Supplementary Figure 4F) were also significantly correlated with the level of certain immune cells. As TMB and MSI were referred as predictive markers for tumor immunotherapy efficacy in cancer (Lin et al., 2021), we also analyzed the correlation between the expression of the prognostic signature gene and the TMB/MSI score. As a result, the TMB score increased as the expression of *MMP* and *SLC22A4* increased (Figure 8A, *p* < 0.05). However, the MSI score was significantly positively correlated with the *MMP* expression, while it was significantly negatively correlated with the *SLC22A4* expression (Figure 8B, *p* < 0.05). In order to clarify the role of prognostic signature genes in the development of ccRCC, we then analyzed the correlation between the expression of these genes and clinical characters. As ccRCC progressed, *MMP9* expression increased, while the expression of *DYSF*, *SELP*, and *KCNJ15* decreased (Figure 9A). Compared with patients with lymphatic node metastasis, ccRCC patients without lymphatic node metastasis had a higher *SLC22A4* expression (Figure 9B). Moreover, ccRCC patients with distant metastasis had a higher *MMP9* expression and a lower expression of *DYSF*, *SLC22A4*, *SELP*, and *KCNJ15* (Figure 9C, all *p* < 0.05). Interestingly, a significant difference was obtained in the expression of *G0S2*, *DYSF*, *MMP9*, *SLC22A4*, *SELP*, and *KCNJ15* between ccRCC patients with high-grade and low-grade tumors (Figure 9D, all *p* < 0.05). A critical process to develop drug scanning biomarker is to analyze the correlation between gene expression and existing therapy targets. In our study, high expression of *SLC22A4*, *G0S2*, and *DYSF*, and low expression of *MMP9* and *SELP* were significantly associated with drug resistance of *CTRP* (Figure 9E). We then constructed a PPI network, and *MMP9* was identified as the hub gene for further analysis among prognostic signature genes (Figure 9F).

### 3.7 lncRNA–miRNA–mRNA regulatory axis

**FIGURE 10:**
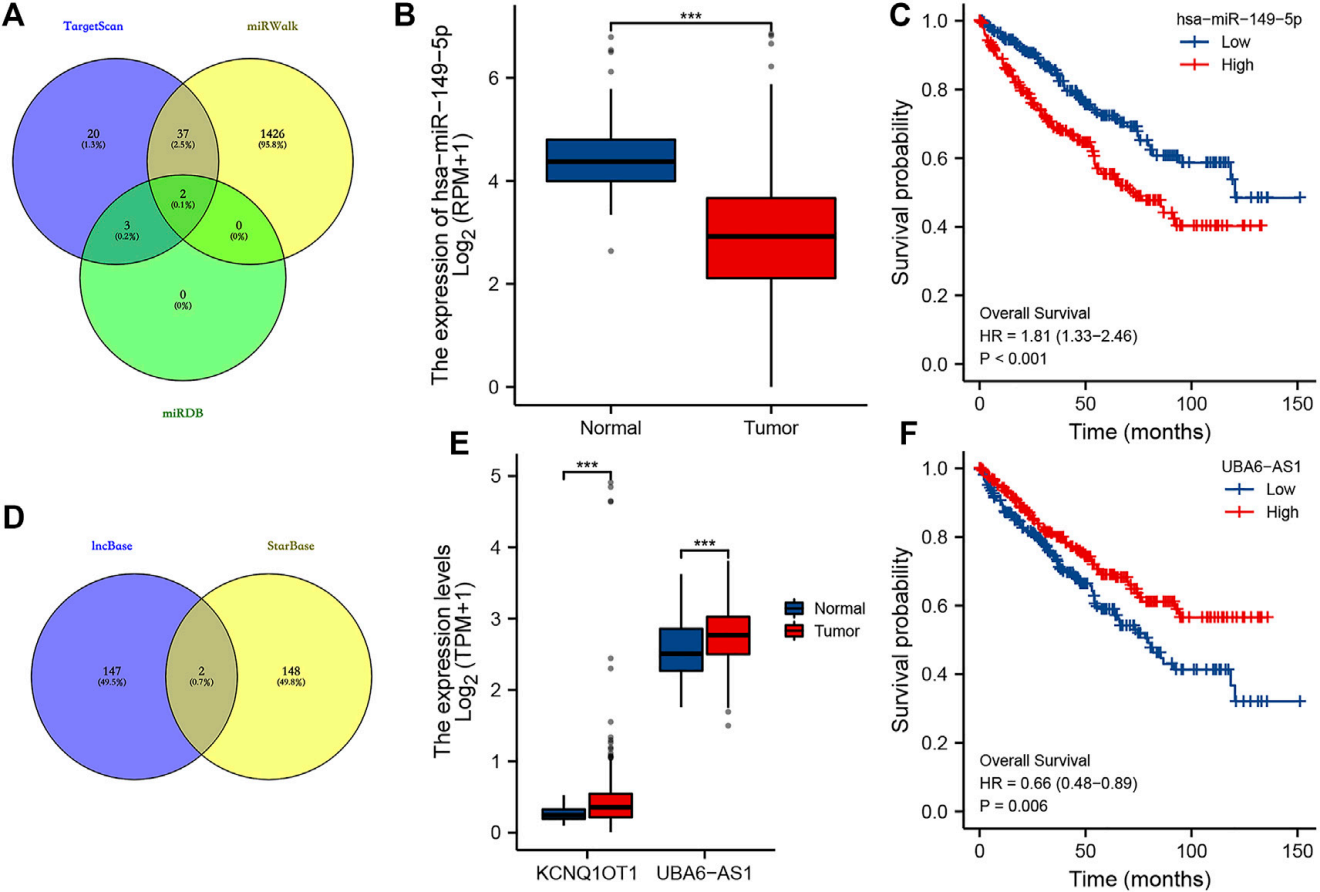
LncRNA–miRNA–mRNA regulatory axis in ccRCC. **(A)** miRNA targets of MMP9 based on the data from TargetScan, miRWalk, and miRDB databases. **(B–C)** Expression and prognostic analysis of the miRNA target in ccRCC. **(D)** LncRNA targets of miR-149-5p based on the data from lncBase and starBase. **(E–F)** Expression and prognostic analysis of the lncRNA target in ccRCC. ***p < 0.001; ccRCC, clear cell renal cell carcinoma.

The lncRNA–miRNA–mRNA regulatory axis played a vital role in the progression of cancer (Wang et al., 2020; Chen et al., 2021; Zhan et al., 2021). We then explored the MMP9-related lncRNA–miRNA–mRNA regulatory axis. Combined with the miRNA targets predicted by TargetScan, miRDB, and miRWalk, miR-6734-3p and miR-149-5p were considered the potential targets of *MMP9* (Figure 10A). Further analysis revealed that miR-149-5p was downregulated in ccRCC (Figure 10B, *p* < 0.001), and high miR-149-5p expression was significantly correlated with a poor OS rate (Figure 10C, *p* < 0.001). Thus, we suggested miR-149-5p as the miRNA target of *MMP9*. Combined with the lncRNA targets predicted by lncBase and starBase, lncRNA KCNQ1OT1 and UBA6-AS1 were considered as the potential targets of miR-149-5p (Figure 10D). Expression analysis revealed that the expression of lncRNA KCNQ1OT1 and UBA6-AS1 was upregulated in ccRCC (Figure 10E, all *p* < 0.001). However, prognosis analysis suggested that only UBA6-AS1 was significantly correlated with the OS rate in ccRCC (Figure 10F, *p* = 0.006). Thus, we suggested UBA6-AS1 as the lncRNA target of miR-149-5p. In conclusion, we identified the lncRNA UBA6-AS1/miR-149-5p/MMP9 regulatory axis for the progression of ccRCC. In our further study, we will focus on the validation of the lncRNA UBA6-AS1/miR-149-5p/MMP9 regulatory axis by *in vivo* and *in vitro* studies.

## 4 Discussion

ccRCC was one of the most common subtypes of urologic neoplasms (D'Avella et al., 2020). Although significant progresses had been made in the therapy of ccRCC, the prognosis of advanced and metastatic patients is still poor (Zeng et al., 2021). No ideal prognostic biomarker or signature has been identified for the prognosis of RCC clinically. Molecular subtype classification of cancer could achieve precise treatment (Kamoun et al., 2020; Zhu et al., 2020). NETs were involved in tumor cell awaking, tumor relapse, and tumor growth and spread (Demkow, 2021). Although several studies had reported the significant role of certain NET-related genes in RCC (Ha et al., 2019), the specific role of NETs in the development and prognosis of RCC has not been fully clarified. Thus, our study was performed.

We first performed consensus clustering analysis based on 23 differentially expressed NET-related genes (*SELPLG*, *LILRB2*, *ITGB2*, *CSF3R*, *ITGAM*, *TLR2*, *CREB5*, *TLR7*, *DYSF*, *TLR8*, *MMP9*, *CYBB*, *PTAFR*, SIGLEC14, *FPR1*, *SLC22A4*, *DNASE1*, *MTOR*, *CYP4F3*, F3, *SELP*, *KCNJ15*, and *G0S2*) in ccRCC. As a result, a total of three clusters of ccRCC were identified. Moreover, cluster 2 had the best OS rate, and cluster 3 had the worst OS rate among these three clusters of ccRCC patients. Further study suggested a lower abundance of immune cells and higher IC_50_ values of gemcitabine, cisplatin, axitinib, sorafenib, and sunitinib in cluster 3 than that in cluster 1/2 in RCC. These pieces of evidence suggested that ccRCC patients in cluster 3 may be more resistant to common chemotherapy, targeted therapy, and immunotherapy. Also, this may be one of the reasons why ccRCC patients in cluster 3 had the worst OS. Based on the result of consensus clustering analysis, we could choose different methods to treat the patients, thus achieving precise treatment. The previous study had highlighted the vital role of consensus clustering and subtype identification in treatment strategies selection (Li et al., 2019).

We then constructed a prognostic signature including six NET-related genes (*G0S2*, *DYSF*, *MMP9*, *SLC22A4*, *SELP*, and *KCNJ15*) for ccRCC. The AUCs of 1-year, 3-year, and 5-year ROC curves were 0.691, 0.692, and 0.699, respectively, suggesting that this prognostic signature had a good performance in predicting the prognosis of ccRCC. Accumulating studies had suggested the important role of NETs in the prognosis of cancers. Naifei et al. constructed a NET-related signature that could predict the prognosis and immunotherapy response in head and neck squamous cell carcinoma (Chen et al., 2022). Another study also developed an innovative prognostic symbol based on the NET-related lncRNA signature for the prognosis in lung cancer (Fang et al., 2021). Another pan-cancer analysis suggested the NET score as a hazardous factor in most cancer types, and a higher score was correlated with more adverse outcomes (Zhang et al., 2022).

We identified the lncRNA UBA6-AS1/miR-149-5p/MMP9 regulatory axis for the progression of ccRCC. The lncRNA UBA6-AS1 could suppress the biological process by inhibiting the decay of UBA6 in ovarian cancer. Moreover, miR-149-5p was involved in cellular migration, proliferation, and apoptosis in RCC (Jin et al., 2016). Another study suggested that miR-149-5p was a prognostic biomarker of ccRCC (Xie et al., 2018). Tianbo et al. suggested MMP9 as a novel biomarker and immunotherapy target for ccRCC, and MMP9 exerted a vital function in tumor immunity (Xu et al., 2021). These pieces of evidence suggested that the lncRNA UBA6-AS1/miR-149-5p/MMP9 may be involved in the progression of ccRCC. In our further study, we will focus on the validation of the lncRNA UBA6-AS1/miR-149-5p/MMP9 regulatory axis by *in vivo* and *in vitro* studies.

There were some limitations to our study. First, it would be better to verify three subtypes of ccRCC using another dataset. Moreover, the lncRNA UBA6-AS1/miR-149-5p/MMP9 regulatory axis should be verified using *in vivo* and *in vitro* studies. Whether genetic characteristics could affect the prognosis of patients with renal cell carcinoma need to be further clarified.

## 5 Conclusion

Collectively, the current study identified three molecular clusters and a prognostic signature for ccRCC based on neutrophil extracellular traps. Integrative transcriptome analyses plus clinical sample validation may facilitate biomarker discovery and clinical transformation.

## Data Availability

The original contributions presented in the study are included in the article/Supplementary Material; further inquiries can be directed to the corresponding author.

## Supplementary material

Supplementary Figure S1 Functional enrichment analysis of NET-related genes. The enriched items in Gene Ontology **(A)** and Kyoto Encyclopedia of Genes and Genomes **(B)** analyses.

Supplementary Figure S2 Prognostic signature in ccRCC based on NET-related genes in the ICGC dataset. **(A)** Riskscore distribution, patients’ survival status, and gene expression profile of the prognostic signature. **(B)** ccRCC patients in the high-risk group had a poor OS rate compared to that in the low-risk group. **(C)** AUCs of 1-year, 3-year, and 5-year ROC curves were 0.623, 0.654, and 0.638, respectively. NETs, neutrophil extracellular traps; ccRCC, clear cell renal cell carcinoma.

Supplementary Figure S3 Correlation between the riskscore and immune cells in ccRCC. The correlation between the riskscore and the level of B cells **(A)**, CD4^+^ T cells **(B)**, CD8^+^ T cells **(C)**, neutrophils **(D)**, macrophages **(E),** and dendritic cells **(F)** in ccRCC.

Supplementary Figure S4 Correlation between prognostic signature genes and immune cells in ccRCC. The correlation between immune cell infiltration and the level of *DYSF* **(A)**, *MMP9* **(B)**, *SLC22A4* **(C)**, *SELP* **(D)**, *KCNJ15* **(E)**, and *G0S2* **(F)** in ccRCC.

## References

[B1] Bi K. He M. X. Bakouny Z. Kanodia A. Napolitano S. Wu J. (2021). Tumor and immune reprogramming during immunotherapy in advanced renal cell carcinoma. Cancer Cell 39 (5), 649–661.e5. e645. 10.1016/j.ccell.2021.02.015 33711272 PMC8115394

[B2] Bonaventura A. Vecchié A. Abbate A. Montecucco F. (2020). Neutrophil extracellular traps and cardiovascular diseases: An update. Cells 9 (1), E231. 10.3390/cells9010231 PMC7016588 31963447

[B3] Chen B. Khodadoust M. S. Liu C. L. Newman A. M. Alizadeh A. A. (2018). Profiling tumor infiltrating immune cells with CIBERSORT. Methods Mol. Biol. 1711, 243–259. 10.1007/978-1-4939-7493-1_12 29344893 PMC5895181

[B4] Chen N. He D. Cui J. (2022). A neutrophil extracellular traps signature predicts the clinical outcomes and immunotherapy response in head and neck squamous cell carcinoma. Front. Mol. Biosci. 9, 833771. 10.3389/fmolb.2022.833771 35252353 PMC8894649

[B5] Chen N. Zhu X. Zhu Y. Shi J. Zhang J. Tang C. (2021). The regulatory relationship and function of LncRNA FAM225A-miR-206-ADAM12 in gastric cancer. Am. J. Transl. Res. 13 (8), 8632–8652. 34539984 PMC8430187

[B6] Chowdhury N. Drake C. G. Kidney Cancer (2020). Kidney cancer: An overview of current therapeutic approaches. Urol. Clin. North Am. 47 (4), 419–431. 10.1016/j.ucl.2020.07.009 33008493

[B7] D'Avella C. Abbosh P. Pal S. K. Geynisman D. M. (2020). Mutations in renal cell carcinoma. Urol. Oncol. 38 (10), 763–773. 10.1016/j.urolonc.2018.10.027 30478013

[B8] Deleuze A. Saout J. Dugay F. Peyronnet B. Mathieu R. Verhoest G. (2020). Immunotherapy in renal cell carcinoma: The future is now. Int. J. Mol. Sci. 21 (7), 2532. 10.3390/ijms21072532 32260578 PMC7177761

[B9] Demkow U. (2021). Neutrophil extracellular traps (NETs) in cancer invasion, evasion and metastasis. Cancers (Basel) 13 (17), 4495. 10.3390/cancers13174495 34503307 PMC8431228

[B10] Fang C. Liu F. Wang Y. Yuan S. Chen R. Qiu X. (2021). A innovative prognostic symbol based on neutrophil extracellular traps (NETs)-related lncRNA signature in non-small-cell lung cancer. Aging (Albany NY) 13 (13), 17864–17879. 10.18632/aging.203289 34257164 PMC8312458

[B11] Gray R. E. Harris G. T. (2019). Renal cell carcinoma: Diagnosis and management. Am. Fam. Physician 99 (3), 179–184. 30702258

[B12] Ha M. Jeong H. Roh J. S. Lee B. Han M. E. Oh S. O. (2019). DYSF expression in clear cell renal cell carcinoma: A retrospective study of 2 independent cohorts. Urol. Oncol. 37 (10), 735–741. 10.1016/j.urolonc.2019.07.007 31377166

[B13] Huang H. Zhang H. Onuma A. E. Tsung A. (2020). Neutrophil elastase and neutrophil extracellular traps in the tumor microenvironment. Adv. Exp. Med. Biol. 1263, 13–23. 10.1007/978-3-030-44518-8_2 32588320 PMC11770835

[B14] Jin L. Li Y. Liu J. Yang S. Gui Y. Mao X. (2016). Tumor suppressor miR-149-5p is associated with cellular migration, proliferation and apoptosis in renal cell carcinoma. Mol. Med. Rep. 13 (6), 5386–5392. 10.3892/mmr.2016.5205 27121091

[B15] Kamoun A. de Reyniès A. Allory Y. Sjödahl G. Robertson A. G. Seiler R. (2020). A consensus molecular classification of muscle-invasive bladder cancer. Eur. Urol. 77 (4), 420–433. 10.1016/j.eururo.2019.09.006 31563503 PMC7690647

[B16] Li B. Cui Y. Nambiar D. K. Sunwoo J. B. Li R. (2019). The immune subtypes and landscape of squamous cell carcinoma. Clin. Cancer Res. 25 (12), 3528–3537. 10.1158/1078-0432.CCR-18-4085 30833271 PMC6571041

[B17] Li J. Xie L. Xie Y. Wang F. (2020). Bregmannian consensus clustering for cancer subtypes analysis. Comput. Methods Programs Biomed. 189, 105337. 10.1016/j.cmpb.2020.105337 31962279

[B18] Li T. Fan J. Wang B. Traugh N. Chen Q. Liu J. S. TIMER (2017). Timer: A web server for comprehensive analysis of tumor-infiltrating immune cells. Cancer Res. 77 (21), e108–e110. 10.1158/0008-5472.CAN-17-0307 29092952 PMC6042652

[B19] Lin W. Chen Y. Wu B. Chen Y. Li Z. (2021). Identification of the pyroptosis-related prognostic gene signature and the associated regulation axis in lung adenocarcinoma. Cell Death Discov. 7 (1), 161. 10.1038/s41420-021-00557-2 34226539 PMC8257680

[B20] Liu J. Zhang S. Dai W. Xie C. Li J. C. (2020). Comprehensive analysis of prognostic value and immune infiltration of chromobox family members in colorectal cancer. Front. Oncol. 10, 582667. 10.3389/fonc.2020.582667 33014884 PMC7498700

[B21] Masucci M. T. Minopoli M. Del Vecchio S. Carriero M. V. (2020). The emerging role of neutrophil extracellular traps (NETs) in tumor progression and metastasis. Front. Immunol. 11, 1749. 10.3389/fimmu.2020.01749 33042107 PMC7524869

[B22] Motzer R. J. Hutson T. E. Tomczak P. Michaelson M. D. Bukowski R. M. Rixe O. (2007). Sunitinib versus interferon alfa in metastatic renal-cell carcinoma. N. Engl. J. Med. 356 (2), 115–124. 10.1056/NEJMoa065044 17215529

[B23] Papayannopoulos V. (2018). Neutrophil extracellular traps in immunity and disease. Nat. Rev. Immunol. 18 (2), 134–147. 10.1038/nri.2017.105 28990587

[B24] Rada B. (2019). Neutrophil extracellular traps. Methods Mol. Biol. 1982, 517–528. 10.1007/978-1-4939-9424-3_31 31172493 PMC6874304

[B25] Riazalhosseini Y. Lathrop M. (2016). Precision medicine from the renal cancer genome. Nat. Rev. Nephrol. 12 (11), 655–666. 10.1038/nrneph.2016.133 27694978

[B26] Ricketts C. J. De Cubas A. A. Fan H. Smith C. C. Lang M. Reznik E. (2018). The cancer genome atlas comprehensive molecular characterization of renal cell carcinoma. Cell Rep. 23 (1), 3698–4326. e315. 10.1016/j.celrep.2018.06.032 29925010

[B27] Şenbabaoğlu Y. Gejman R. S. Winer A. G. Liu M. Van Allen E. M. de Velasco G. (2016). Erratum to: Tumor immune microenvironment characterization in clear cell renal cell carcinoma identifies prognostic and immunotherapeutically relevant messenger RNA signatures. Genome Biol. 17 (1), 46. 10.1186/s13059-017-1180-8 27855702 PMC5114739

[B28] Shang B. Guo L. Shen R. Cao C. Xie R. Jiang W. (2021). Prognostic significance of NLR about NETosis and lymphocytes perturbations in localized renal cell carcinoma with tumor thrombus. Front. Oncol. 11, 771545. 10.3389/fonc.2021.771545 34993135 PMC8724023

[B29] Su D. Singer E. A. Srinivasan R. (2015). Molecular pathways in renal cell carcinoma: Recent advances in genetics and molecular biology. Curr. Opin. Oncol. 27 (3), 217–223. 10.1097/CCO.0000000000000186 25811348

[B30] Vorobjeva N. V. Chernyak B. V. (2020). NETosis: Molecular mechanisms, role in physiology and Pathology. Biochemistry. 85 (10), 1178–1190. 10.1134/S0006297920100065 33202203 PMC7590568

[B31] Wang J. Y. Yang Y. Ma Y. Wang F. Xue A. Zhu J. (2020). Potential regulatory role of lncRNA-miRNA-mRNA axis in osteosarcoma. Biomed. Pharmacother. 121, 109627. 10.1016/j.biopha.2019.109627 31810120

[B32] Wilkerson M. D. Hayes D. N. (2010). ConsensusClusterPlus: A class discovery tool with confidence assessments and item tracking. Bioinformatics 26 (12), 1572–1573. 10.1093/bioinformatics/btq170 20427518 PMC2881355

[B33] Xie M. Lv Y. Liu Z. Zhang J. Liang C. Liao X. (2018). Identification and validation of a four-miRNA (miRNA-21-5p, miRNA-9-5p, miR-149-5p, and miRNA-30b-5p) prognosis signature in clear cell renal cell carcinoma. Cancer Manag. Res. 10, 5759–5766. 10.2147/CMAR.S187109 30532596 PMC6245347

[B34] Xu T. Gao S. Liu J. Huang Y. Chen K. Zhang X. (2021). MMP9 and IGFBP1 regulate tumor immune and drive tumor progression in clear cell renal cell carcinoma. J. Cancer 12 (8), 2243–2257. 10.7150/jca.48664 33758602 PMC7974879

[B35] Yipp B. G. Kubes P. (2013). NETosis: How vital is it? Blood 122 (16), 2784–2794. 10.1182/blood-2013-04-457671 24009232

[B36] Zeng Q. Sun S. Li Y. Li X. Li Z. Liang H. (2019). Identification of therapeutic targets and prognostic biomarkers among CXC chemokines in the renal cell carcinoma microenvironment. Front. Oncol. 9, 1555. 10.3389/fonc.2019.01555 32117786 PMC7012904

[B37] Zeng X. Zhu C. Zhu X. (2021). DUSP4 promotes the carcinogenesis of CCRCC via negative regulation of autophagic death. Biosci. Biotechnol. Biochem. 85 (8), 1839–1845. 10.1093/bbb/zbab111 34143206

[B38] Zhan T. Gao X. Wang G. Li F. Shen J. Lu C. (2021). Construction of novel lncRNA-miRNA-mRNA network associated with recurrence and identification of immune-related potential regulatory Axis in hepatocellular carcinoma. Front. Oncol. 11, 626663. 10.3389/fonc.2021.626663 34336642 PMC8320021

[B39] Zhang Y. Guo L. Dai Q. Shang B. Xiao T. Di X. (2022). A signature for pan-cancer prognosis based on neutrophil extracellular traps. J. Immunother. Cancer 10 (6), e004210. 10.1136/jitc-2021-004210 35688556 PMC9189842

[B40] Zhou L. Li Y. Li Z. Huang Q. (2020). Mining therapeutic and prognostic significance of STATs in renal cell carcinoma with bioinformatics analysis. Genomics 112 (6), 4100–4114. 10.1016/j.ygeno.2020.06.032 32640276

[B41] Zhu S. Yu W. Yang X. Wu C. Cheng F. (2020). Traditional classification and novel subtyping systems for bladder cancer. Front. Oncol. 10, 102. 10.3389/fonc.2020.00102 32117752 PMC7025453

