# Supplementary figures and images for: Identification and validation of the molecular subtype and prognostic signature for clear cell renal cell carcinoma based on neutrophil extracellular traps
Source: Front Cell Dev Biol. 2022 Nov 29;10:1021690. doi: 10.3389/fcell.2022.1021690 (PMC9745193; doi:10.3389/fcell.2022.1021690)

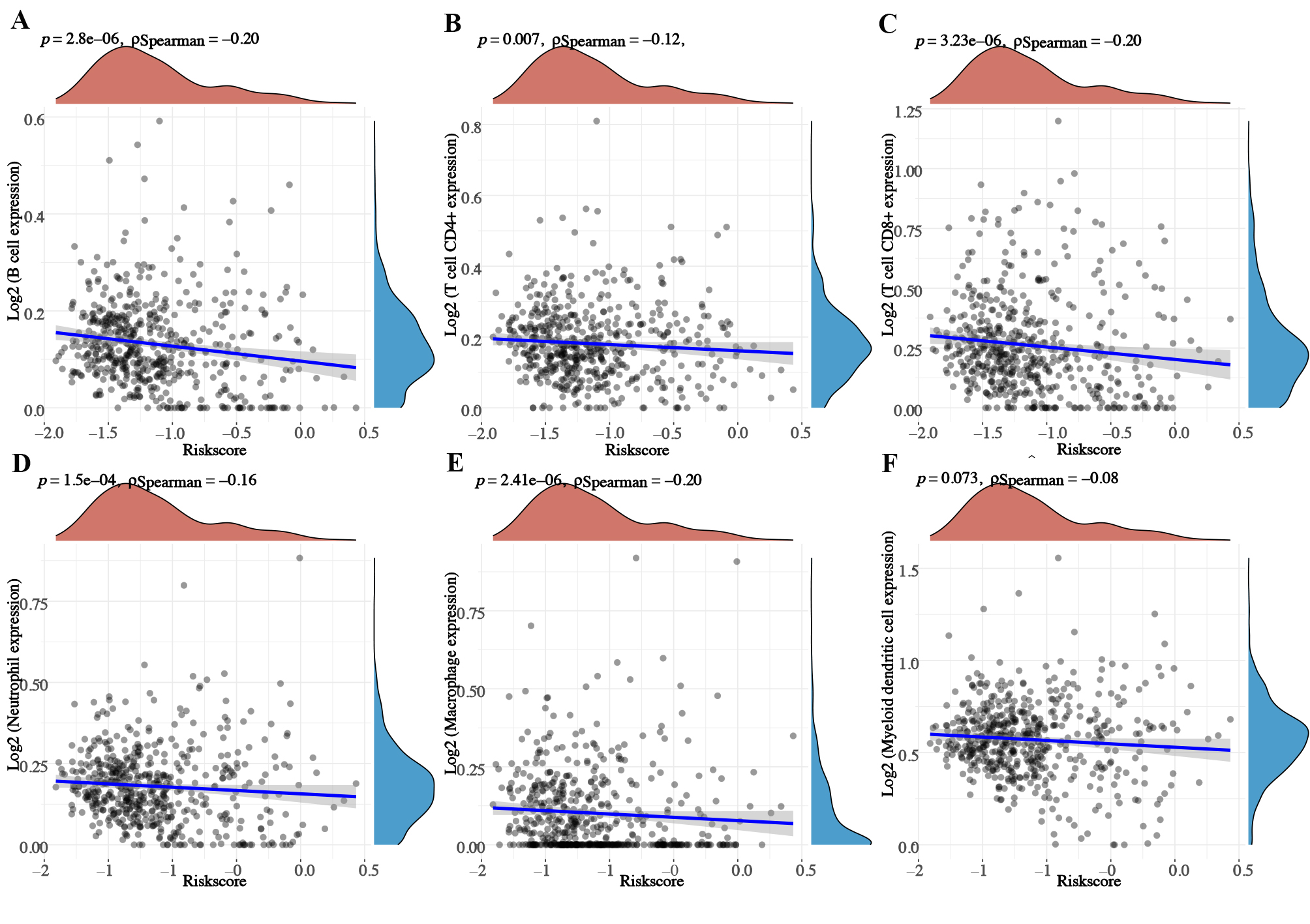

Supplement: Supplementary file 1 [file Image3.JPEG]

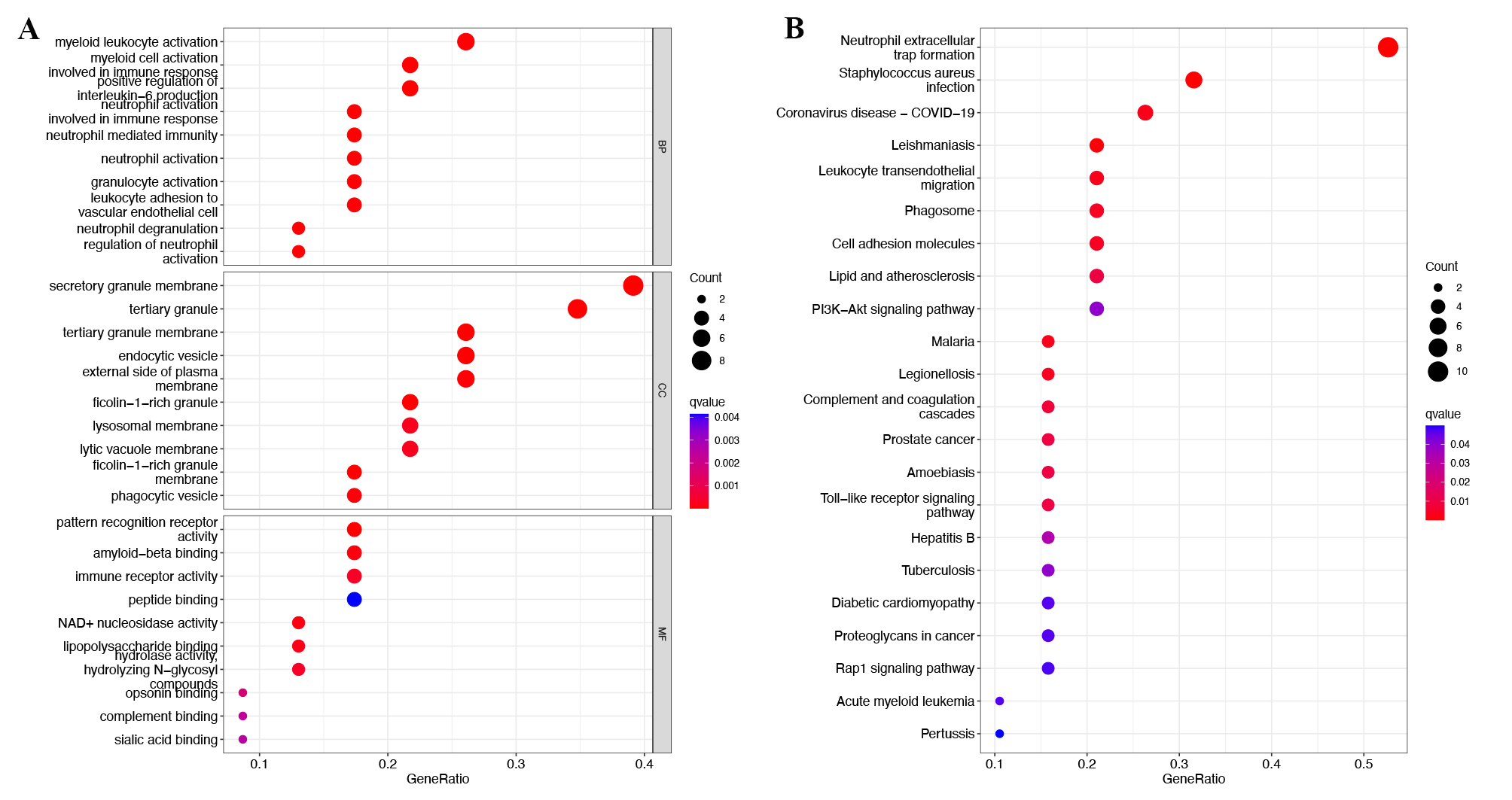

Supplement: Supplementary file 3 [file Image1.JPEG]

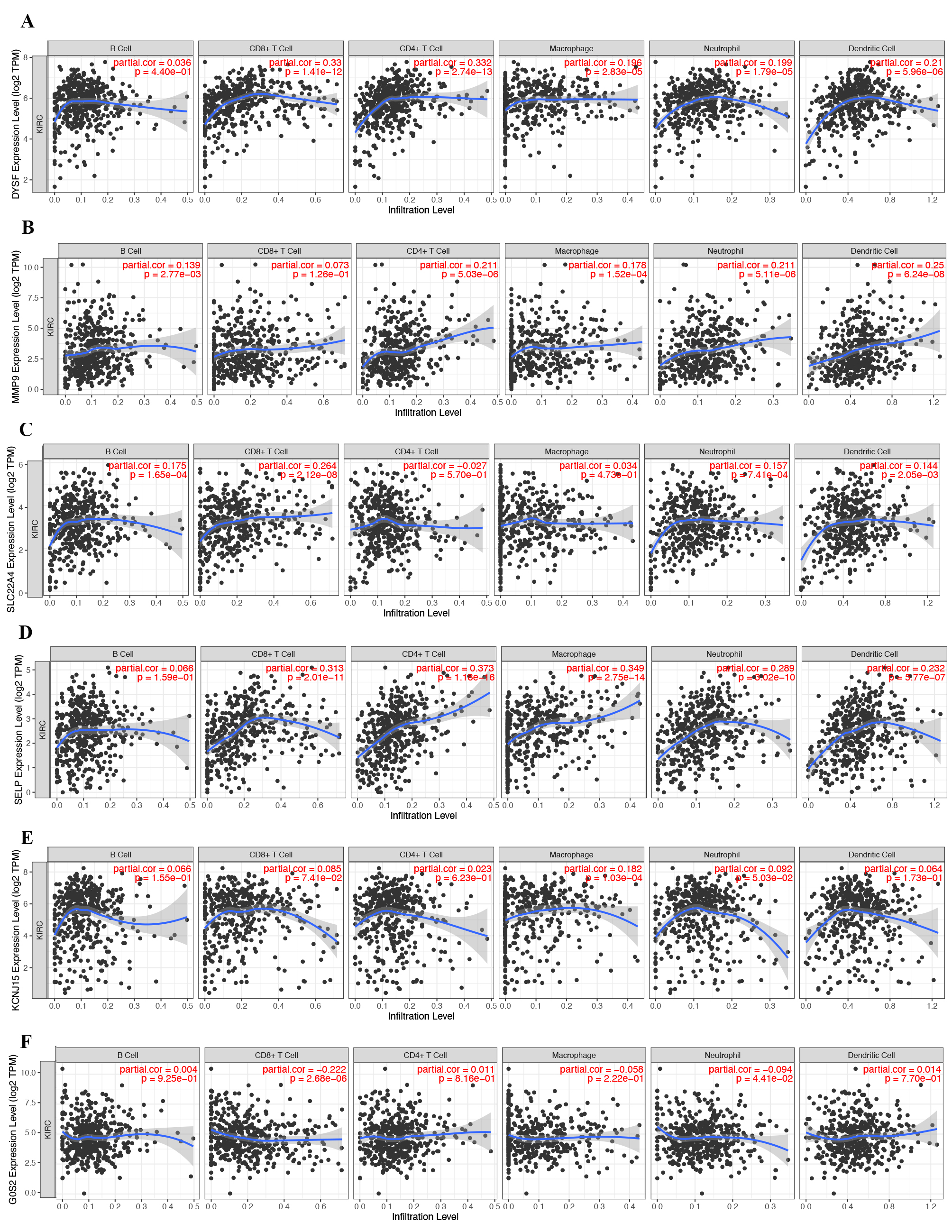

Supplement: Supplementary file 4 [file Image4.JPEG]

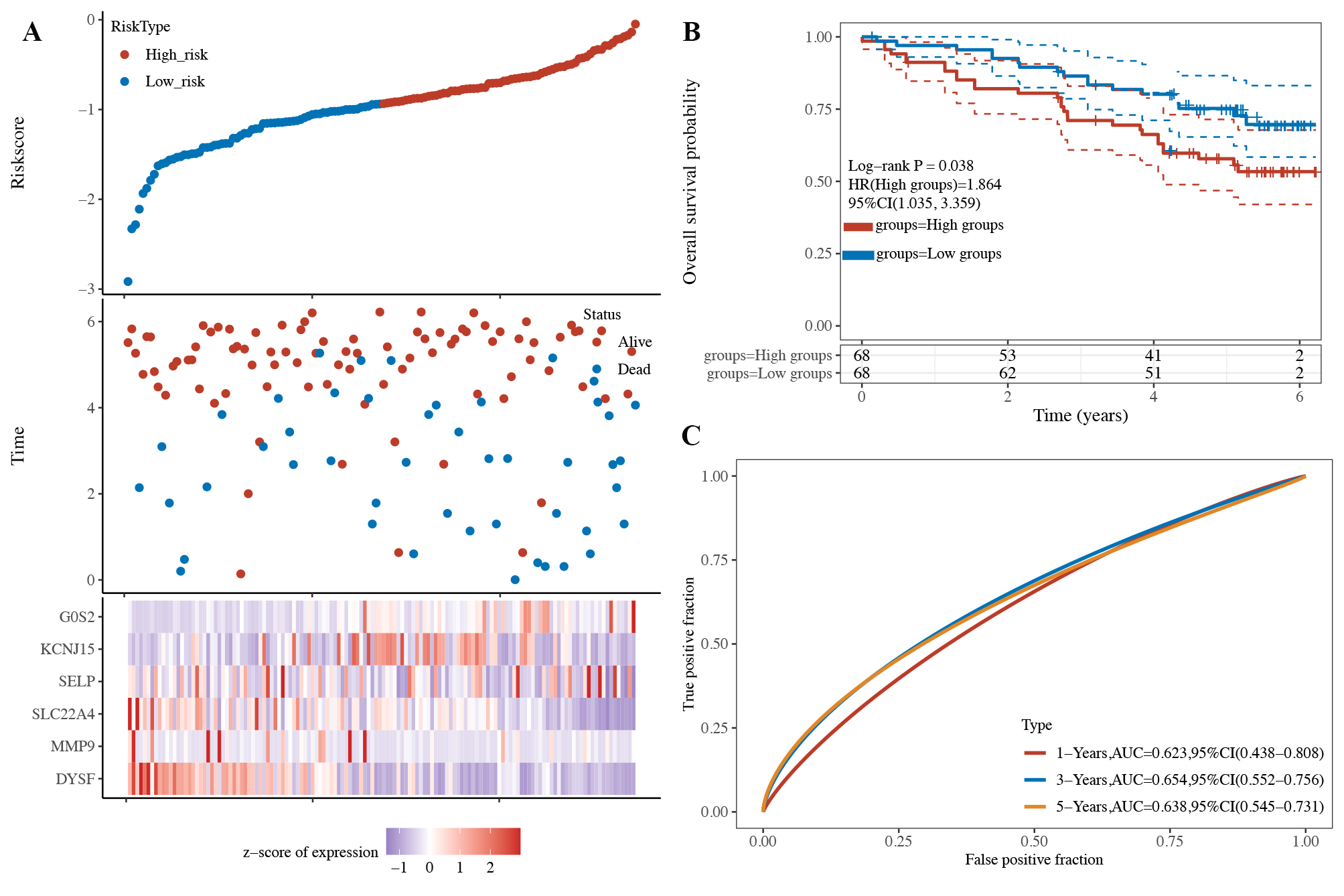

Supplement: Supplementary file 6 [file Image2.JPEG]
