# Supplementary material for: Identification and validation of the molecular subtype and prognostic signature for clear cell renal cell carcinoma based on neutrophil extracellular traps
Source: Front Cell Dev Biol. 2022 Nov 29;10:1021690. doi: 10.3389/fcell.2022.1021690 (PMC9745193; doi:10.3389/fcell.2022.1021690)
Supplement: Supplementary file 2 [file Table1.DOCX]

Supplementary table 1. Neutrophil extracellular traps-related genes obtained from previous study

| HGNC symbol | Entrez Gene | Ensembl | Description (coding protein) |
| --- | --- | --- | --- |
| ALPL | 249 | ENSG00000162551 | Alkaline phosphatase, biomineralization associated |
| BST1 | 683 | ENSG00000109743 | Bone marrow stromal cell antigen 1 |
| CD93 | 22918 | ENSG00000125810 | CD93 antigen |
| CEACAM3 | 1084 | ENSG00000170956 | Carcinoembryonic antigen-related cell adhesion molecule 3 |
| CREB5 | 9586 | ENSG00000146592 | Cyclic AMP-responsive element-binding protein 5 |
| CRISPLD2 | 83716 | ENSG00000103196 | Cysteine rich secretory protein LCCL domain containing |
| CSF3R | 1441 | ENSG00000119535 | Colony stimulating factor 3 receptor |
| CYP4F3 | 4051 | ENSG00000186529 | Cytochrome P450 family 4 subfamily F member 3 |
| DYSF | 8291 | ENSG00000135636 | Dysferlin |
| FCAR | 2204 | ENSG00000186431 | Fc fragment of IgA receptor |
| FCGR3B | 2215 | ENSG00000162747 | Fc fragment of IgG receptor IIIb |
| CPPED1 | 55313 | ENSG00000103381 | Calcineurin like phosphoesterase domain containing |
| FPR1 | 2357 | ENSG00000171051 | Formyl peptide receptor 1 |
| FPR2 | 2358 | ENSG00000171049 | Formyl peptide receptor 2 |
| G0S2 | 50486 | ENSG00000123689 | G0/G1 switch 2 |
| HIST1H2BC | 8347 | ENSG00000180596 | H2B clustered histone 4 |
| HPSE | 10855 | ENSG00000173083 | Heparanase |
| CXCR1 | 3577 | ENSG00000163464 | C-X-C motif chemokine receptor 1 |
| CXCR2 | 3579 | ENSG00000180871 | C-X-C motif chemokine receptor 2 |
| KCNJ15 | 3772 | ENSG00000157551 | Potassium inwardly rectifying channel subfamily J member 15 |
| LILRB2 | 10288 | ENSG00000131042 | Leukocyte immunoglobulin like receptor B2 |
| MGAM | 8972 | ENSG00000257335 | Maltase-glucoamylase |
| MME | 4311 | ENSG00000196549 | Membrane metalloendopeptidase |
| PDE4B | 5142 | ENSG00000184588 | Phosphodiesterase 4B |
| S100A12 | 6283 | ENSG00000163221 | S100 calcium binding protein 2 |
| SIGLEC5 | 8778 | ENSG00000105501 | Sialic acid binding Ig like lectin 5 |
| SLC22A4 | 6583 | ENSG00000197208 | Solute carrier family 22 member 4 |
| SLC25A37 | 51312 | ENSG00000147454 | Solute carrier family 25 member 37 |
| TECPR2 | 9895 | ENSG00000196663 | Tectonin beta-propeller repeat containing 2 |
| TNFRSF10C | 8794 | ENSG00000173535 | TNF receptor superfamily member 10c |
| VNN3 | 55350 | ENSG0000093134 | Vascular non-inflammatory molecule 3 |
| AKT1 | 207 | ENSG00000142208 | AKT serine/threonine kinase 1 |
| AKT2 | 208 | ENSG00000105221 | AKT serine/threonine kinase 2 |
| ATG7 | 10533 | ENSG00000197548 | Autophagy related 7 |
| CLEC6A | 93978 | ENSG00000205846 | Dectin-2 |
| CSF3 | 1440 | ENSG00000108342 | Granulocyte colony stimulating factor |
| CTSG | 1511 | ENSG00000100448 | Cathepsin G |
| CYBB | 1536 | ENSG00000165168 | NADPH oxidase |
| DNASE1 | 1773 | ENSG00000213938 | Deoxyribonuclease I |
| ELANE | 1991 | ENSG00000197561 | Neutrophil elastase |
| ENTPD4 | 14573 | ENSG00000197217 | Ectonucleoside Triphosphate Diphosphohydrolase 4 |
| F3 | 2152 | ENSG00000117525 | Coagulation Factor III, tissue factor |
| HMGB1 | 3146 | ENSG00000189403 | High mobility group box 1 |
| IL17A | 3605 | ENSG00000112115 | Interleukin 17 |
| IL1B | 3553 | ENSG00000125538 | Interleukin 1 beta |
| IL6 | 3569 | ENSG00000136244 | Interleukin 6 |
| IL8 | 3576 | ENSG00000169429 | Interleukin 8 |
| IRAK4 | 51135 | ENSG00000198001 | Interleukin 1 receptor associated kinase 4 |
| ITGAM | 3684 | ENSG00000169896 | Complement component 3 receptor 3 subunit |
| ITGB2 | 3689 | ENSG00000160255 | Complement component 3 receptor 3 and 4 subunit |
| KCNN3 | 3782 | ENSG00000143603 | Potassium channel, calcium activated |
| MAPK1 | 5594 | ENSG00000100030 | Mitogen-activated protein kinase 1 |
| MAPK3 | 5595 | ENSG00000102882 | Mitogen-activated protein kinase 3 |
| MMP9 | 4218 | ENSG00000100985 | Matrix metallopeptidase 9 |
| MPO | 4353 | ENSG00000005381 | Myeloperoxidase |
| MTOR | 2475 | ENSG00000198793 | Mechanistic target of rapamycin kinase |
| PADI4 | 23569 | ENSG00000159339 | Peptidyl arginine deiminase 4 |
| PTAFR | 5724 | ENSG00000169403 | Platelet activation factor receptor |
| PIK3CA | 5290 | ENSG00000121879 | Phosphatidylinositol-4,5-bisphosphate 3-kinase |
| RIPK1 | 8737 | ENSG00000137275 | Receptor interacting serine/threonine kinase 1 |
| RIPK3 | 11035 | ENSG00000129465 | Receptor interacting serine/threonine kinase 3 |
| SELP | 6403 | ENSG00000174175 | P-selectin |
| SELPLG | 6404 | ENSG00000110876 | P-selectin receptor |
| SIGLEC14 | 10049587 | ENSG00000254415 | Sialic acid binding Ig like lectin 4 |
| TLR2 | 7097 | ENSG00000137462 | Toll like receptor 2 |
| TLR4 | 7099 | ENSG00000136869 | Toll like receptor 4 |
| TLR7 | 51284 | ENSG00000196664 | Toll like receptor 7 |
| TLR8 | 51311 | ENSG00000101916 | Toll like receptor 8 |
| TNF | 7124 | ENSG00000232810 | Tumor necrosis factor-alpha |
